# Risk of surgical site infection, acute kidney injury, and *Clostridium difficile* infection following antibiotic prophylaxis with vancomycin plus a beta-lactam versus either drug alone: A national propensity-score-adjusted retrospective cohort study

**DOI:** 10.1371/journal.pmed.1002340

**Published:** 2017-07-10

**Authors:** Westyn Branch-Elliman, John E. Ripollone, William J. O’Brien, Kamal M. F. Itani, Marin L. Schweizer, Eli Perencevich, Judith Strymish, Kalpana Gupta

**Affiliations:** 1 Department of Medicine, VA Boston Healthcare System, West Roxbury, Massachusetts, United States of America; 2 Harvard Medical School, Boston, Massachusetts, United States of America; 3 VA Center for Healthcare Organization and Implementation Research, VA Boston Healthcare System, West Roxbury, Massachusetts, United States of America; 4 Boston University School of Public Health, Boston, Massachusetts, United States of America; 5 Boston University School of Medicine, Boston, Massachusetts, United States of America; 6 Department of Surgery, VA Boston Healthcare System, West Roxbury, Massachusetts, United States of America; 7 VA Comprehensive Access & Delivery Research & Evaluation, Iowa City VA Health Care System, Iowa City, Iowa, United States of America; 8 Carver College of Medicine, University of Iowa, Iowa City, Iowa, United States of America; University College London, UNITED KINGDOM

## Abstract

**Background:**

The optimal regimen for perioperative antimicrobial prophylaxis is controversial. Use of combination prophylaxis with a beta-lactam plus vancomycin is increasing; however, the relative risks and benefits associated with this strategy are unknown. Thus, we sought to compare postoperative outcomes following administration of 2 antimicrobials versus a single agent for the prevention of surgical site infections (SSIs). Potential harms associated with combination regimens, including acute kidney injury (AKI) and *Clostridium difficile* infection (CDI), were also considered.

**Methods and findings:**

Using a multicenter, national Veterans Affairs (VA) cohort, all patients who underwent cardiac, orthopedic joint replacement, vascular, colorectal, and hysterectomy procedures during the period from 1 October 2008 to 30 September 2013 and who received planned manual review of perioperative antimicrobial prophylaxis regimen and manual review for the 30-day incidence of SSI were included. Using a propensity-adjusted log-binomial regression model stratified by type of surgical procedure, the association between receipt of 2 antimicrobials (vancomycin plus a beta-lactam) versus either single agent alone (vancomycin or a beta-lactam) and SSI was evaluated. Measures of association were adjusted for age, diabetes, smoking, American Society of Anesthesiologists score, preoperative methicillin-resistant *Staphylococcus aureus* (MRSA) status, and receipt of mupirocin. The 7-day incidence of postoperative AKI and 90-day incidence of CDI were also measured. In all, 70,101 procedures (52,504 beta-lactam only, 5,089 vancomycin only, and 12,508 combination) with 2,466 (3.5%) SSIs from 109 medical centers were included. Among cardiac surgery patients, combination prophylaxis was associated with a lower incidence of SSI (66/6,953, 0.95%) than single-agent prophylaxis (190/12,834, 1.48%; crude risk ratio [RR] 0.64, 95% CI 0.49, 0.85; adjusted RR 0.61, 95% CI 0.46, 0.83). After adjusting for SSI risk, no association between receipt of combination prophylaxis and SSI was found for the other types of surgeries evaluated, including orthopedic joint replacement procedures. In MRSA-colonized patients undergoing cardiac surgery, SSI occurred in 8/346 (2.3%) patients who received combination prophylaxis versus 4/100 (4.0%) patients who received vancomycin alone (crude RR 0.58, 95% CI 0.18, 1.88). Among MRSA-negative and -unknown cardiac surgery patients, SSIs occurred in 58/6,607 (0.9%) patients receiving combination prophylaxis versus 146/10,215 (1.4%) patients who received a beta-lactam alone (crude RR 0.61, 95% CI 0.45, 0.83). Based on these associations, the number needed to treat to prevent 1 SSI in MRSA-colonized patients is estimated to be 53, compared to 176 in non-MRSA patients. CDI incidence was similar in both exposure groups. Across all types of surgical procedures, risk of AKI was increased in the combination antimicrobial prophylaxis group (2,971/12,508 [23.8%] receiving combination versus 1,058/5,089 [20.8%] receiving vancomycin alone versus 7,314/52,504 [13.9%] receiving beta-lactam alone). We found a significant association between absolute risk of AKI and receipt of combination regimens across all types of procedures. If the observed association is causal, the number needed to harm for severe AKI following cardiac surgery would be 167. The major limitation of our investigation is that it is an observational study in a predominantly male population, which may limit generalizability and lead to unmeasured confounding.

**Conclusions:**

There are benefits but also unintended consequences of antimicrobial and infection prevention strategies aimed at “getting to zero” healthcare-associated infections. In our study, combination prophylaxis was associated with both benefits (reduction in SSIs following cardiac surgical procedures) and harms (increase in postoperative AKI). In cardiac surgery patients, the difference in risk–benefit profile by MRSA status suggests that MRSA-screening-directed prophylaxis may optimize benefits while minimizing harms in this selected population. More information about long-term outcomes and patient and societal preferences regarding risk of SSI versus risk of AKI is needed to improve clinical decision-making.

## Abstract 2

In a large observational study, Kalpana Gupta and colleagues investigate the benefits and risks of antibiotic prophylaxis for surgical procedures.

## Author summary

**Why was this study done?:**

- Surgical site infections are a costly and highly morbid postoperative complication that can be prevented with preoperative antibiotic prophylaxis. Use of single-drug antibiotic prophylaxis is recommended in clinical guidelines, but increasing rates of antibiotic-resistant infections have raised the question of whether antibiotic prophylaxis regimens should be expanded to better cover resistant organisms.
- The goal of this study was to determine if expanded antimicrobial prophylaxis regimens—with 2 antibiotics rather than the standard 1—are associated with reductions in surgical site infections. A second goal was to determine if use of expanded antimicrobial prophylaxis results in other harms, such as increases in postoperative *Clostridium difficile* infection or acute kidney injury.

**What did the researchers do and find?:**

- Study investigators compared the association between receipt of expanded antimicrobial prophylaxis and the rate of surgical site infections, *Clostridium difficile* infections, and acute kidney injury in a national United States veteran population.
- The study found that expanded prophylaxis was associated with a reduction in surgical site infections following cardiac surgical procedures but not following other types of surgeries.
- Expanded prophylaxis was associated with an increase in postoperative acute kidney injury after all types of surgeries evaluated. The magnitude of the risk of surgical site infection was similar to that of severe acute kidney injury.
- There was no association between receipt of expanded prophylaxis and *Clostridium difficile* infection.

**What do these findings mean?:**

- These findings suggest that expanded prophylaxis is associated with both benefits (reduction in surgical site infections following cardiac surgical procedures) and harms (increase in postoperative acute kidney injury).
- The risks and harms of expanded prophylaxis for reducing surgical site infections should be weighed before implementing this intervention on a large scale. More information about long-term outcomes and patient and societal preferences about risk of surgical site infection versus risk of acute kidney injury is needed to better inform clinical decision-making about expanded versus standard surgical prophylaxis.

## Introduction

The optimal perioperative antimicrobial regimen for the prevention of surgical site infections (SSIs) remains an open question. The multidisciplinary *Clinical Practice Guidelines for Antimicrobial Prophylaxis in Surgery*, a combined endorsement of the American Society of Health-System Pharmacists, Infectious Diseases Society of America, Surgical Infection Society, and the Society for Healthcare Epidemiology of America, recommends a single agent—most often a beta-lactam antibiotic—for most surgical procedures [1]. While routine use of vancomycin is not recommended, prophylaxis with vancomycin as a single agent is a consideration in methicillin-resistant *Staphylococcus aureus* (MRSA)–colonized patients and in institutions with a high incidence of MRSA infections [1]. A large meta-analysis suggested that beta-lactams are as effective—if not more so—than glycopeptide antibiotics, including vancomycin, in patients undergoing cardiac surgery [2].

In the setting of increasing incidence of MRSA infections and SSI, the optimal surgical prophylaxis regimen was called into question [3]. A large Veterans Affairs (VA)–based cohort study suggested that MRSA colonization status may be a risk modifier for optimal antimicrobial prophylaxis regimen: MRSA-colonized patients had a lower incidence of SSI if they received vancomycin, whereas MRSA-negative or -unknown patients had fewer SSIs after receipt of a beta-lactam antibiotic [4]. Modeling also suggested that vancomycin is the preferred agent in cardiac surgery patients when the institutional prevalence of MRSA colonization exceeds 3% [5].

However, due to reports of increasing incidence of methicillin-susceptible *S*. *aureus* (MSSA) SSI after receipt of vancomycin alone for surgical prophylaxis [6], guidelines note that a combined regimen of vancomycin and a beta-lactam (combination therapy) may be considered in settings where MSSA and MRSA infections are both commonly encountered [1]. Regimens containing beta-lactams may also be preferred when gram-negative enteric organisms (Enterobacteriaceae) are major SSI pathogens. Despite these recommendations, there are very few published data on the relative benefits and harms of combination versus single antimicrobial prophylaxis for SSI prevention and the impact on microbiological outcomes.

One small, retrospective, single-center study evaluated single versus combination antimicrobial prophylaxis for patients undergoing total joint replacement procedures and found a marginal benefit to the 2-drug regimen [7]. A larger study suggested a benefit of combination antimicrobial prophylaxis for MRSA-colonized patients undergoing joint replacement surgeries [8]. However, this study was primarily designed to evaluate a bundle of interventions and thus could not isolate the effect of combination versus single antimicrobial therapy. Further, only MRSA-colonized patients received combination therapy. Thus, the benefit of combination therapy versus monotherapy remains unclear, particularly as a practice strategy for patients undergoing clean surgical procedures, where the morbidity associated with SSI can be severe, regardless of MRSA status.

On the other hand, despite a potential benefit of combination antimicrobial prophylaxis in terms of SSI prevention, other investigations have demonstrated that implementation of combination prophylaxis is potentially harmful: A recent study demonstrated significantly higher rates of acute kidney injury (AKI) after total joint replacement amongst patients who received combination antimicrobial prophylaxis compared to cefazolin alone [9]. In addition, recent reports suggest that combination regimens with vancomycin and a beta-lactam may increase AKI in other hospital settings [10]. Increasing incidence of *Clostridium difficile* infection (CDI) after receipt of combination therapy is also a theoretical concern [11].

Given these outstanding questions, our objective was to test the hypothesis that combination prophylaxis regimens with vancomycin plus a beta-lactam are associated with a lower incidence of SSI compared to single-agent prophylaxis. In this large, multicenter, national cohort of VA surgical patients at 109 facilities, we evaluated both the potential benefits and the unintended consequences of this intervention.

## Methods

### Ethics statement

This study was approved by the VA Boston Healthcare System institutional review board (IRB) (#2770) prior to data collection and was reviewed by the VA’s Surgical Quality Data Use Group (SQDUG) and External Peer Review Program (EPRP) committees prior to final submission.

### Database development

The national cohort included surgical patients who were evaluated by the EPRP, which includes manual abstraction of type, time of administration, and duration of surgical antibiotic prophylaxis regimen, and by the VA Surgical Quality Improvement Program (VASQIP), where a nurse reviewer, trained in VASQIP methodology, manually reviews 30-day endpoints that include SSI categorized as superficial, deep, or organ space, using recent Centers for Disease Control and Prevention National Healthcare Safety Network definitions. The superficial SSI outcome was available for non-cardiac procedures for the entire study period, but only for fiscal year 2012–2013 for the cardiac procedures.

EPRP has a standard method for identifying Surgical Care Improvement Program (SCIP) cases for surgical review, as outlined in the fiscal year 2012 EPRP technical manual. VASQIP utilizes a validated sampling methodology for manual review that limits high-volume operations, such as inguinal hernia repairs. This results in approximately 30% of all VA surgical cases being abstracted by the nurse reviewers, with these abstractions comprising 70% of all major cases [12].

**Fig 1:**
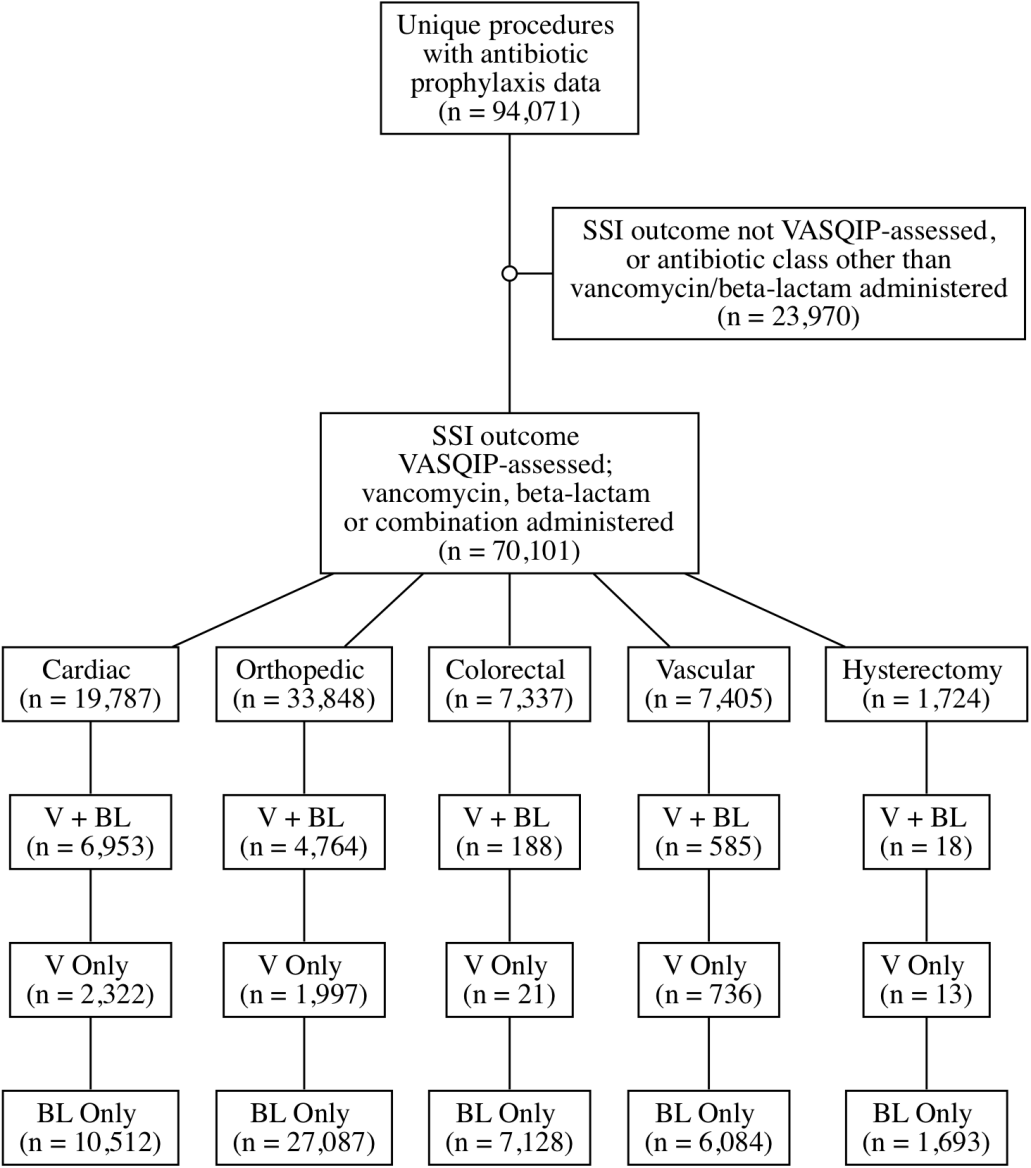
Creation of cohort: Eligible surgeries and distribution by surgical prophylaxis regimen. BL, beta lactam; SSI, surgical site infection; V, vancomycin; VASQIP, Veterans Affairs Surgical Quality Improvement Program.

Patients who underwent cardiac (bypass procedures, valve repairs, and repair of structural heart disease), orthopedic joint (knee or hip) replacement, vascular (peripheral and central bypass and shunting procedures, arterial and vein resection procedures, and endarterectomies), colorectal, or hysterectomy procedures and received a beta-lactam, vancomycin, or both during the period from 1 October 2008 to 30 September 2013 were eligible for inclusion in the cohort (Fig 1; S1 Table). Surgical prophylaxis regimen, MRSA preoperative screening, mupirocin decolonization, and preoperative chlorhexidine are driven by local clinical practices rather than any universal protocol applied across the national VA healthcare system. Patients were not eligible if prophylaxis data were not available, if they received an agent other than a beta-lactam or vancomycin (either alone or in combination with either of these agents), or if they had a duplicate record.

Covariates collected from the surgical databases were chosen a priori and were based on published risk factors for our outcomes of interest; these included age, diabetes, current smoking, and American Society of Anesthesiologists (ASA) score. Mupirocin orders and laboratory results, including microbiology and *C*. *difficile* results, were extracted from the VA Corporate Data Warehouse. Additional variables extracted from VA administrative databases included facility complexity, facility region, and facility volume.

All methodology, including data analysis, was conducted using an a priori IRB-approved proposal dated 8 September 2013 (S1 IRB Protocol). Data analyses, including propensity adjustments, regression techniques, and stratification by surgical type and MRSA colonization status, were preplanned. Facility was evaluated as a potential instrumental variable but did not meet criteria, and thus no further instrumental variable analysis was attempted. During creation of the cohort, several studies were published suggesting a potential association between combination antimicrobial regimens containing vancomycin and a beta-lactam and increased incidence of AKI. Given these reports, a secondary analysis was added to elucidate this relationship.

### Primary outcome

The primary outcome was SSI within 30 days of surgery, determined by the VASQIP manual review process [12]. The primary exposure evaluated was surgical prophylaxis with combination therapy (vancomycin plus a beta-lactam) versus either drug alone. Due to inherent differences in surgical prophylaxis by surgical type, the primary analysis was stratified by procedure, consistent with our a priori analysis plan. All SSI microbiology results are presented as descriptive and unadjusted analyses.

### Secondary analyses

#### MRSA colonization status

MRSA colonization was defined as any positive microbiological culture for MRSA within the 30 days prior to surgery or positive MRSA PCR nasal screen within 30 days preoperatively and/or on the day of surgery. If a record did not have a positive PCR or culture for MRSA, it was classified as negative in the primary analysis. The MRSA-negative group was further stratified by known negative (negative MRSA result available) and unknown (no MRSA result identified). Evaluation of combination versus single-agent prophylaxis was also stratified by MRSA colonization status. In MRSA-negative patients, a beta-lactam alone was the reference exposure, and for MRSA-colonized patients, vancomycin was the reference exposure.

#### Adverse outcomes

The association between receipt of 2 drugs versus 1 drug and the postoperative incidence of AKI (any AKI and AKI stages 1, 2, and 3 as defined by the Acute Kidney Injury Network [13]) within 7 days was evaluated. CDI was defined as any positive *C*. *difficile* PCR, toxin, or culture within 90 days following surgery.

### Data analysis

Crude and adjusted binomial models with log-link functions were constructed to estimate risk ratios (RRs) corresponding to the association of combination therapy versus either antibiotic (cardiac, orthopedic, and vascular procedures) or an individual antibiotic (beta-lactam alone for hysterectomies and colorectal procedures, where vancomycin alone is specifically not recommended by clinical guidelines) with all outcomes. Crude risk ratios (cRRs) and adjusted risk ratios (aRRs) are presented. Crude and adjusted binomial models with identity link functions were constructed to estimate risk differences. Values for number needed to treat (NNT) and number needed to harm (NNH) were generated by calculating the reciprocals of the adjusted absolute risk difference and corresponding 95% confidence interval limits.

All adjusted models were constructed using the pure propensity score corresponding to the probability of receiving combination therapy. Propensity scores were generated via logistic regression. A separate set of propensity scores was constructed for each type of procedure using the set of covariates appropriate for the corresponding outcome model (SSI, AKI, or CDI). For the SSI outcome, the covariates included age, diabetes status, ASA score, receipt of mupirocin, smoking status, and MRSA colonization (if not already stratified by MRSA status). For the AKI outcome, the set included age, diabetes status, ASA score, smoking status, and prophylaxis regimen duration. For the CDI outcome, the set included all variables used for the SSI outcome and specific surgery type. A maximum of 0.3% of observations were deleted from any of these logistic regression models due to missing outcome or explanatory variable data. Within each type of procedure, all cases with propensity score values below the 2.5th percentile or above the 97.5th percentile of the propensity score distribution were trimmed to increase the comparability of propensity score distributions between the exposed and unexposed groups, in accordance with accepted methods [14,15]. If any RR model did not converge, a Poisson distribution replaced the binomial distribution in the model specification, and robust standard errors were calculated [16–18]. All analyses were performed in SAS version 9.2 (SAS Institute, Cary, North Carolina).

## Results

During the period from 1 October 2008 to 30 September 2013, 70,101 eligible surgical cases at 109 unique facilities nationwide received perioperative prophylaxis with a beta-lactam only (52,504), vancomycin only (5,089), or both (12,508) (Fig 1). In total, 2,466 (3.5%) SSIs were identified across all surgical types. Preoperative MRSA colonization was identified in 2,527 (3.6%) patients. Baseline demographics are presented in Table 1.

**Table 1:** Variable distributions among all VASQIP-assessed surgeries.

Characteristic	Full cohort, *n* = 70,101	V + BL, *n* = 12,508	V only, *n* = 5,089	BL only, *n* = 52,504
**Age, mean (SD), years**	64.2 (9.8)	64.7 (9.0)	64.5 (9.0)	64.0 (10.0)
**Male**	66,005 (94.16%)	12,145 (97.10%)	4,862 (95.54%)	48,998 (93.32%)
**Race**^a^				
American Indian	454 (0.65%)	75 (0.60%)	35 (0.69%)	344 (0.66%)
Asian	208 (0.30%)	55 (0.44%)	18 (0.35%)	135 (0.26%)
Black	10,141 (14.47%)	1,913 (15.29%)	698 (13.72%)	7,530 (14.34%)
Hawaiian/Pacific Islander	388 (0.55%)	97 (0.78%)	20 (0.39%)	271 (0.52%)
White	54,281 (77.43%)	9,544 (76.30%)	3,978 (78.17%)	40,759 (77.63%)
Unknown	4,604 (6.57%)	821 (6.56%)	338 (6.64%)	3,445 (6.56%)
**Ethnicity**^a^				
Hispanic	3,447 (4.92%)	674 (5.39%)	205 (4.03%)	2,568 (4.89%)
Non-Hispanic	63,976 (91.26%)	11,328 (90.57%)	4,686 (92.08%)	47,962 (91.35%)
Unknown	2,653 (3.78%)	503 (4.02%)	196 (3.85%)	1,954 (3.72%)
**Surgery type**				
Cardiac	19,787 (28.23%)	6,953 (55.59%)	2,322 (45.63%)	10,512 (20.02%)
Orthopedic	33,848 (48.28%)	4,764 (38.09%)	1,997 (39.24%)	27,087 (51.59%)
Colorectal	7,337 (10.47%)	188 (1.50%)	21 (0.41%)	7,128 (13.58%)
Vascular	7,405 (10.56%)	585 (4.68%)	736 (14.46%)	6,084 (11.59%)
Hysterectomy	1,724 (2.46%)	18 (0.14%)	13 (0.26%)	1,693 (3.22%)
**Diabetes**^a^	18,825 (26.85%)	3,986 (31.87%)	1,582 (31.09%)	13,257 (25.25%)
**Current smoker**^a^	20,438 (29.16%)	3,311 (26.47%)	1,543 (30.32%)	15,584 (29.68%)
**ASA score > 2**^a^	58,401 (83.31%)	11,074 (88.54%)	4,476 (87.95%)	42,851 (81.61%)
**Mupirocin administered**	6,749 (9.63%)	1,991 (15.92%)	684 (13.44%)	4,074 (7.76%)
**Preoperative MRSA colonization**^b^				
Positive	2,527 (3.60%)	666 (5.32%)	326 (6.41%)	1,535 (2.92%)
Negative	48,171 (68.72%)	8,010 (64.04%)	3,201 (62.90%)	36,960 (70.39%)
Not tested/unknown	19,403 (27.68%)	3,832 (30.64%)	1,562 (30.69%)	14,009 (26.68%)
**SSI (any)**^b^	2,466 (3.52%)	214 (1.71%)	120 (2.36%)	2,132 (4.06%)
***C*. *difficile* infection**^b^	558 (0.80%)	90 (0.72%)	48 (0.94%)	420 (0.80%)
**AKI (any)**^a^^**,**^^b^^**,**^^c^	11,343 (16.18%)	2,971 (23.75%)	1,058 (20.79%)	7,314 (13.93%)
Stage 1	9,394 (13.40%)	2,464 (19.70%)	865 (17.00%)	6,065 (11.55%)
Stage 2	1,292 (1.84%)	329 (2.63%)	122 (2.40%)	841 (1.60%)
Stage 3	657 (0.94%)	178 (1.42%)	71 (1.40%)	408 (0.78%)

Data are given as n (percent) unless otherwise indicated.

aPercent missing: race, 0.03%; ethnicity, 0.03%; diabetes, <0.00%; current smoker, 0.01%; ASA, 0.01%; AKI (any), 11.05%.

bDefinitions and time frames as outlined in text.

cStage 1 AKI was defined as an increase in serum creatinine ≥ 26.5 μmol/l (0.3 mg/dl) and/or an increase to 1.5- to <2.0-fold the baseline level. Stage 2 AKI was defined as an increase in serum creatinine to 2.0- to <3.0-fold the baseline level, and Stage 3 AKI was defined as an increase of serum creatinine to ≥3.0-fold the baseline level or an increase in creatinine from <353.6 μmol/l (4.0 mg/dl) to ≥353.6 μmol/l (4.0 mg/dl) with at least a 44.2-μmol/l (0.5-mg/dl) rise.

AKI, acute kidney injury; ASA, American Society of Anesthesiologists; BL, beta-lactam; MRSA, methicillin-resistant Staphylococcus aureus; SSI, surgical site infection; V, vancomycin.

### Primary analyses

In patients undergoing cardiac surgery, 66/6,953 (0.95%) patients receiving combination prophylaxis developed SSI versus 190/12,834 (1.48%) patients receiving single-agent prophylaxis (cRR 0.64, 95% CI 0.49, 0.85; NNT 189). After controlling for age, diabetes, ASA score, mupirocin administration, current smoking status, and preoperative MRSA colonization status, receipt of combination antimicrobial prophylaxis was associated with reduced SSI risk following cardiac surgical procedures (Table 2; aRR 0.61, 95% CI 0.46, 0.83; NNT 176). When combination therapy was compared to either of the single agents alone, the associations were similar (beta-lactam alone: cRR 0.64, 95% CI 0.48, 0.85; aRR 0.61, 95% CI 0.45, 0.83; vancomycin alone: cRR 0.67, 95% CI 0.44, 1.01; aRR 0.65, 95% CI 0.43, 0.99).

**Table 2:** Results of propensity-score-based analyses of the effect of antibiotic regimen on 30-day surgical site infection incidence.

Outcome	Number with outcome	Crude risk, exposed	Crude risk, unexposed^a^	RR (95% CI)	RD (95% CI)	NNT (95% CI)^b^
***Cardiac***						
Crude (*n =* 19,787)	256	66/6,953 (0.95%)	190/12,834 (1.48%)	0.64 (0.49, 0.85)	−0.005 (−0.008, −0.002)	189 (120, 455)
PS adjusted (*n =* 18,624)	230			0.61 (0.46, 0.83)	−0.006 (−0.009, −0.003)	176 (114, 385)
*MRSA colonized*						
Crude (*n =* 446)	12	8/346 (2.31%)	4/100 (4.00%)	0.58 (0.18, 1.88)	−0.017 (−0.058, 0.025)	
PS adjusted (*n =* 421)	11			0.53 (0.16, 1.77)^c^	−0.019 (−0.091, 0.052)	
*MRSA−/unknown*						
Crude (*n =* 16,822)	204	58/6,607 (0.88%)	146/10,215 (1.43%)	0.61 (0.45, 0.83)	−0.006 (−0.009, −0.002)	182 (115, 435)
PS adjusted (*n =* 15,989)	191			0.60 (0.43, 0.82)	−0.006 (−0.009, −0.002)	176 (110, 417)
***Orthopedic***						
Crude (*n =* 33,848)	435	68/4,764 (1.43%)	367/29,084 (1.26%)	1.13 (0.87, 1.46)	0.002 (−0.002, 0.005)	
PS adjusted (*n =* 31,860)	382			1.09 (0.82, 1.44)	0.001 (−0.003, 0.005)	
*MRSA colonized*						
Crude (*n =* 426)	13	9/262 (3.44%)	4/164 (2.44%)	1.41 (0.44, 4.50)	0.010 (−0.022, 0.042)	
PS adjusted (*n =* 399)	10			1.21 (0.31, 4.75)	0.015 (−0.024, 0.055)	
*MRSA−/unknown*						
Crude (*n =* 30,908)	371	59/4,502 (1.31%)	312/26,406 (1.18%)	1.11 (0.84, 1.46)	0.001 (−0.002, 0.005)	
PS adjusted (*n =* 29,368)	347			1.08 (0.81, 1.45)	0.001 (−0.003, 0.005)	
***Vascular***						
Crude (*n =* 7,405)	606	50/585 (8.55%)	556/6,820 (8.15%)	1.05 (0.79, 1.38)	0.004 (−0.020, 0.028)	
PS adjusted (*n =* 6,882)	542			0.99 (0.74, 1.33)	−0.001 (−0.024, 0.023)	
*MRSA colonized*						
Crude (*n =* 96)	11	3/40 (7.50%)	8/56 (14.29%)	0.53 (0.15, 1.86)	−0.068 (−0.191, 0.055)	
PS adjusted (*n =* 87)	10			0.71 (0.20, 2.53)	0.057 (−0.199, 0.312)	
*MRSA−/unknown*						
Crude (*n =* 6,388)	508	47/545 (8.62%)	461/5,843 (7.89%)	1.09 (0.82, 1.46)	0.007 (−0.017, 0.030)	
PS adjusted (*n =* 5,944)	465			1.02 (0.75, 1.38)	0.001 (−0.023, 0.026)	
***Colorectal***						
Crude (*n =* 7,316)	1,112	30/188 (15.96%)	1,082/7,128 (15.18%)	1.05 (0.75, 1.47)	0.008 (−0.045, 0.061)	
PS adjusted (*n =* 6,695)	1,022			1.07 (0.76, 1.50)	0.013 (−0.043, 0.069)	

Via the pure propensity score, all models were adjusted for age, diabetes status, ASA score, mupirocin status, and smoking status. The full cohort models also were adjusted for MRSA colonization status.

aReference group was either antibiotic alone for the cardiac, orthopedic, and vascular full cohort models; vancomycin alone for MRSA-colonized-only models; and beta-lactam alone for MRSA−/unknown and colorectal models.

bA NNT value was not applicable if the CI for the risk difference crossed 1.

cPoisson distribution with log link and robust standard errors from generalized estimating equations applied.

ASA, American Society of Anesthesiologists; MRSA, methicillin-resistant Staphylococcus aureus; NNT, number needed to treat; PS, propensity score; RD, risk difference; RR, risk ratio.

There was no association between SSI reduction and combination regimens for the other types of surgical procedures assessed. This was true for the orthopedic procedures (68/4,764 [1.43%] with combination versus 367/29,084 [1.26%] with single agent; cRR 1.13, 95% CI 0.87, 1.46; aRR 1.09, 95% CI 0.82, 1.44) and for the vascular surgery procedures (50/585 [8.55%] with combination versus 556/6,820 [8.15%] with single agent; cRR 1.05, 95% CI 0.79, 1.38; aRR 0.99, 95% CI 0.74, 1.33). There was also no benefit of combination regimens for colorectal procedures (Table 2) or for hysterectomies (0/18 SSIs in combination therapy group, 48/1,693 in the beta-lactam only group, Fisher’s exact *p*-value = 1.0); in both of these cases, patients receiving vancomycin alone were excluded based on recommended clinical practices. For full details, including unadjusted numbers and proportions, see Table 2.

The model risk reduction estimates were similar when separating superficial from deep/organ-space SSI (S2 Table). Adjusting for facility-level variables, including facility complexity, region, and volume, did not affect the findings, regardless of surgical type (S3 Table).

### Secondary analyses

#### MRSA colonization status and surgical site infections

In a bivariate model, MRSA colonization was associated with a higher risk of SSI across all surgery types (173/2,527 in MRSA colonized versus 2,293/6,754 in MRSA negative or unknown; cRR 2.02, 95% CI 1.74, 2.34).

Among MRSA-colonized patients undergoing cardiac surgery, SSI occurred in 8/346 (2.3%) patients receiving combination therapy versus 4/100 (4%) receiving vancomycin alone (Table 2; aRR 0.53, 95% CI 0.16, 1.77).

In MRSA-negative or -unknown patients undergoing cardiac surgery, SSI occurred in 58/6,607 (0.88%) receiving combination prophylaxis compared to 146/10,215 (1.4%) receiving beta-lactam alone (aRR 0.60, 95% CI 0.43, 0.82; Table 2). Exclusion of MRSA-unknown patients (i.e., restricting the cohort to confirmed MRSA-negative patients) yielded similar associations (aRR 0.55, 95% CI 0.38, 0.80). In MRSA-unknown patients, there were 46 SSIs; the aRR of combination therapy compared to beta-lactam was 0.83 (95% CI 0.45, 1.52).

Among MRSA-colonized patients undergoing cardiac surgery, the associated absolute risk reduction for SSI was approximately triple that of the absolute risk reduction in MRSA-negative or -unknown patients, with a NNT to prevent 1 SSI of 53 for the MRSA-colonized group compared to 176 for the MRSA-negative or -unknown group.

Stratification by MRSA status did not change the lack of association between SSI reduction and combination versus single drug therapy for orthopedic or vascular surgery procedures (Table 2).

#### Microbiology of surgical site infections

Of the 1,279 SSIs with a microbiology result, MRSA was isolated in 101 (7.9%), MSSA in 308 (24.1%), coagulase-negative staphylococci in 150 (12.0%), gram-negative enteric organisms in 512 (40%), and other gram-negative bacteria, mixed growths not otherwise characterized, or no growth in 208 (16.2%).

Among MRSA-colonized cardiac surgery patients, similar numbers of MRSA infections were identified in the beta-lactam only group (3/297, 1.0%), the vancomycin only group (0/100, 0.0%), and the combination prophylaxis group (2/346, 0.60%). MSSA infections were found in 1/346 (0.3%) patients receiving combination prophylaxis, 3/297 (1.0%) patients receiving a beta-lactam alone, and 3/100 (3.0%) patients receiving vancomycin alone.

Among cardiac surgery patients who were not MRSA colonized during the preoperative period, the incidence of SSI was considerably lower. MRSA SSI was found in 0/4,919 (0%) patients who received combination prophylaxis, 3/7,613 (0.04%) patients who received a beta-lactam alone, and 0/1,434 (0%) patients who received vancomycin alone. Incidence of MSSA SSI was 6/4,919 (0.12%) in patients receiving combination therapy, 22/7,613 (0.29%) in patients receiving a beta-lactam, and 3/1,434 (0.21%) in patients receiving vancomycin. SSI due to gram-negative enteric organisms occurred in 38/17,465 (0.22%) patients receiving a beta-lactam-containing regimen compared to 9/2,322 (0.39%) patients receiving vancomycin alone.

In orthopedic surgery patients, MRSA SSI occurred in 9/4,764 (0.19%) patients receiving combination prophylaxis, 27/27,087 (0.10%) receiving a beta-lactam alone, and 1/1,997 (0.05%) receiving vancomycin alone. MSSA SSI incidence was similar across all prophylactic regimens (combination prophylaxis, 100/27,087, 0.37%; beta-lactam 16/14,764, 0.34%; vancomycin 9/1,997, 0.45%). SSI due to coagulase-negative staphylococci occurred in 3/4,764 (0.063%) patients who received a combination regimen, 29/27,087 (0.11%) patients who received a beta-lactam alone, and 1/1,997 (0.50%) patients who received vancomycin alone. The frequency of SSI caused by gram-negative enteric organisms was 42/31,851 (0.13%) in the beta-lactam-containing groups compared to 5/1,997 (0.25%) in the vancomycin only group.

In vascular surgery patients, the rate of MRSA infection was 2/585 (0.34%) in patients receiving combination prophylaxis, 28/6,084 (0.46%) in patients who received a beta-lactam alone, and 1/736 (0.14%) in patients who received vancomycin alone. MSSA SSI occurred in 11/585 (1.9%) patients receiving combination prophylaxis, 72/6,084 (1.2%) patients receiving a beta-lactam alone, and 8/736 (1.1%) patients receiving vancomycin alone. Gram-negative enteric SSI occurred in 1.8% of the patients who received any beta-lactam-containing regimen versus 1.4% in patients who received vancomycin alone.

#### *C*. *difficile* infection and acute kidney injury

The rate of CDI was 90/12,508 (0.72%) in patients receiving combination prophylaxis and 468/57,593 (0.81%) in patients receiving any single agent. No association between CDI and prophylaxis regimen was identified (aRR for combination versus single agent 1.01, 95% CI 0.78, 1.31; Table 3).

**Table 3:** Results of propensity-score-based analyses of the effect of antibiotic regimen on secondary outcome incidence.

Outcome	Number with outcome	Crude risk, exposed	Crude risk, unexposed^a^	RR (95% CI)	RD (95% CI)	NNH (95% CI)^b^
**AKI, cardiac surgery**						
Any, crude (*n =* 19,330)	5,791	2,315/6,825 (33.92%)	3,476/12,505 (27.80%)	1.22 (1.17, 1.27)	0.061 (0.048, 0.075)	17 (14, 22)
Any, PS adjusted (*n =* 17,872)	5,283			1.18 (1.12, 1.23)	0.047 (0.033, 0.061)	22 (17, 31)
Stage 1, crude (*n =* 18,430)	4,891	1,943/6,453 (30.11%)	2,948/11,977 (24.61%)	1.22 (1.17, 1.28)	0.055 (0.041, 0.069)	19 (15, 25)
Stage 1, PS adjusted (*n =* 17,084)	4,495			1.19 (1.13, 1.25)	0.044 (0.030, 0.059)	23 (18, 34)
Stage 2, crude (*n =* 14,134)	595	240/4,750 (5.05%)	355/9,384 (3.78%)	1.34 (1.14, 1.57)	0.013 (0.005, 0.020)	79 (50, 186)
Stage 2, PS adjusted (*n =* 13,114)	525			1.19 (1.00, 1.41)	0.006 (−0.001, 0.013)	
Stage 3, crude (*n =* 13,844)	305	132/4,642 (2.84%)	173/9,202 (1.88%)	1.51 (1.21, 1.89)	0.010 (0.004, 0.015)	105 (66, 244)
Stage 3, PS adjusted (*n =* 12,852)	263			1.39 (1.09, 1.77)	0.006 (0.001, 0.012)	167 (87, 2,000)
**AKI, orthopedic joint replacement surgery**						
Any, crude (*n =* 28,332)	3,170	474/3,909 (12.13%)	2,696/24,423 (11.04%)	1.10 (1.00, 1.20)	0.011 (−0.0001, 0.022)	
Any, PS adjusted (*n =* 26,726)	2,978			1.12 (1.02, 1.23)	0.013 (0.002, 0.025)	76 (41, 527)
Stage 1, crude (*n =* 27,779)	2,617	387/3,822 (10.13%)	2,230/23,957 (9.31%)	1.09 (0.98, 1.21)	0.008 (−0.002, 0.018)	
Stage 1, PS adjusted (*n =* 26,205)	2,457			1.11 (1.00, 1.23)	0.010 (−0.001, 0.021)	
Stage 2, crude (*n =* 25,546)	384	57/3,492 (1.63%)	327/22,054 (1.48%)	1.10 (0.83, 1.46)	0.002 (−0.003, 0.006)	
Stage 2, PS adjusted (*n =* 24,111)	363			1.15 (0.86, 1.53)	0.002 (−0.003, 0.007)	
Stage 3, crude (*n =* 25,331)	169	30/3,465 (0.87%)	139/21,866 (0.64%)	1.36 (0.92, 2.02)	0.002 (−0.001, 0.006)	
Stage 3, PS adjusted (*n =* 23,906)	158			1.39 (0.92, 2.09)	0.002 (−0.001, 0.006)	
**AKI, vascular surgery**						
Any, crude (*n =* 7,088)	1,187	131/575 (22.78%)	1,056/6,513 (16.21%)	1.41 (1.20, 1.65)	0.066 (0.030, 0.101)	16 (10, 34)
Any, PS adjusted (*n =* 6,386)	1,064			1.25 (1.04, 1.50)	0.041 (0.003, 0.080)	25 (13, 334)
Stage 1, crude (*n =* 6,852)	951	97/541 (17.93%)	854/6,311 (13.53%)	1.33 (1.09, 1.60)	0.044 (0.011, 0.077)	23 (13, 95)
Stage 1, PS adjusted (*n =* 6,178)	856			1.18 (0.95, 1.47)	0.030 (−0.010, 0.061)	
Stage 2, crude (*n =* 6,045)	144	23/467 (4.93%)	121/5,578 (2.17%)	2.27 (1.47, 3.51)	0.028 (0.008, 0.048)	37 (22, 132)
Stage 2, PS adjusted (*n =* 5,452)	130			1.89 (1.15, 3.09)	0.020 (−0.001, 0.042)	
Stage 3, crude (*n =* 5,993)	92	11/455 (2.42%)	81/5,538 (1.46%)	1.65 (0.89, 3.08)	0.010 (−0.005, 0.024)	
Stage 3, PS adjusted (*n =* 5,400)	78			1.32 (0.65, 2.69)	0.006 (−0.009, 0.021)	
***C*. *difficile* infection, all surgeries**						
Crude (*n =* 70,101)	558	90/12,508 (0.72%)	468/57,593 (0.81%)	0.89 (0.71, 1.11)	−0.001 (−0.003, 0.001)	
PS adjusted (*n =* 57,084)	352			1.01 (0.78, 1.31)	0.000 (−0.002, 0.002)	

Via the pure propensity score, all AKI models were adjusted for age, diabetes status, ASA score, smoking status, and prophylaxis regimen duration and the C. difficile infection model was adjusted for age, diabetes status, ASA score, mupirocin status, smoking status, MRSA colonization status, and specific surgery type. Reference outcome level for all AKI models was no AKI. Observations with unknown AKI status were not used in these analyses.

aExposure reference group was either antibiotic for all models.

bA NNH value was not applicable if the CI for the risk difference crossed 1.

AKI, acute kidney injury; ASA, American Society of Anesthesiologists; MRSA, methicillin-resistant Staphylococcus aureus; NNH, number needed to harm; PS, propensity score; RD, risk difference; RR, risk ratio.

In contrast, combination versus single prophylaxis was associated with higher relative risk of AKI in the 7-day postoperative period, after adjusting for prophylaxis regimen duration, age, diabetes, ASA score, and smoking (Table 3). The adjusted relative risk increase for any AKI associated with receipt of combination therapy ranged from 11% following orthopedic joint replacement procedures to 89% following vascular surgical procedures (for full results of AKI analysis, see Table 3). Findings were similar regardless of whether the comparator agent was vancomycin alone, beta-lactam alone, or either one alone. The majority of patients had Stage 1 AKI, but among the smaller group that had Stage 2 or 3 AKI, the relative risk associated with receipt of combination therapy was higher than for Stage 1 AKI. Overall, the NNH to cause 1 episode of AKI in cardiac surgery patients receiving combination therapy was 22, and, for stage 3 AKI, 167. The NNH associated with 1 additional episode of any postoperative AKI after receipt of combination therapy was 76 following orthopedic procedures and 25 following vascular surgical procedures (Table 3).

## Discussion

Overall, we found that administration of combination prophylaxis was associated with a reduction in SSIs following cardiac surgical procedures, but not following other types of surgical procedures. The risk reduction in cardiac surgery after receipt of combination prophylaxis was found regardless of which single agent was the comparator and was also independent of mupirocin receipt. Preoperative MRSA colonization status affected the baseline risk of SSI and the absolute risk reduction associated with receipt of combination prophylaxis, but not the relative risk reduction associated with this strategy. Furthermore, we demonstrated that although protective against SSI for cardiac procedures, receipt of combination prophylaxis was also associated with an increase in postoperative AKI across all types of surgeries evaluated, independent of which single agent was the comparator and also independent of duration of antimicrobial prophylaxis. In other words, the increase in AKI incidence was not simply associated with the addition of vancomycin to a beta-lactam but also with the addition of a beta-lactam to vancomycin.

To our knowledge, this is the largest national, multicenter cohort study to evaluate not only the potential benefit of combination prophylaxis for MRSA-colonized patients undergoing clean and clean/contaminated surgeries but also the potential harms of this approach. The detailed and pragmatic clinical cohort also facilitated an investigation into the impact of prophylaxis independent of other factors that may mitigate SSI risk, including receipt of mupirocin [19].

Other large cohort studies have suggested an association between reduced SSI rates and expanded antimicrobial prophylaxis regimens following cardiac surgery, with effect estimates ranging from 0.85 (95% CI 0.74, 0.97) to 0.74 (95% CI 0.62, 0.87), depending upon which antimicrobial combinations were used [20]. A large quasi-experimental study evaluating a bundled approach to SSI prevention, including 2 antimicrobial agents for MRSA-colonized patients, found an association with reduced complex *S*. *aureus* SSI in orthopedic patients. However, no benefit was found when SSIs caused by gram-negative bacteria or any pathogen were considered, similar to the findings in our investigation [8]. Although both cardiac and orthopedic procedures are classified as “clean surgeries,” prior studies suggest that the microbiology of the 2 may be different, with more SSIs attributable to coagulase-negative staphylococci following orthopedic procedures and more SSIs caused by gram-negative agents such as gram-negative enteric organisms following cardiac procedures [21–23]. Other studies of combination prophylaxis versus single-drug prophylaxis (primarily beta-lactams) in patients undergoing total joint replacement procedures did not find a reduction in SSI rates when 2 drugs were used for antimicrobial prophylaxis, similar to our findings [7,9]. Furthermore, a recent single-center evaluation of patients undergoing orthopedic implant surgery found no reduction in SSI risk among patients who received cefazolin in addition to vancomycin; however, a significant increase in postoperative AKI was reported [9]. Our findings expand these concepts with a national-level, multicenter cohort, adjusted for preoperative MRSA status and receipt of mupirocin—2 common elements of SSI reduction bundles—and also evaluating multiple adverse postoperative events.

Few studies have evaluated the unintended consequences of the quest to “get to zero” healthcare-associated infections [24]. Previous studies primarily focused on SSI reduction without reporting adverse outcomes associated with broader-spectrum and combined prophylaxis, such as CDI and AKI. In our study, universally across all surgery types evaluated, combination prophylaxis was associated with an increased risk of AKI. Although unmeasured confounding and exposure to other nephrotoxins not accounted for in our analysis may partially explain these results, the findings are consistent with previous studies demonstrating an increase in AKI among patients receiving combination prophylaxis [10]. We found no increased risk of CDI in patients who received combination prophylaxis compared to a single agent, supporting other studies that have evaluated this question [25,26].

These findings can be utilized to optimize SSI reduction while reducing harms such as severe AKI. For cardiac surgery patients, if the observed associations are causal, the NNT to prevent 1 SSI (53) in MRSA-colonized patients was substantially lower than the NNT for MRSA-negative and -unknown patients (176). Given the NNH to cause 1 episode of Stage 3 AKI (167), the risk–benefit ratio apparently shifts from favoring combination prophylaxis for MRSA-positive patients to more potential harm than benefit for MRSA-negative and MRSA-unknown patients. Thus, clinicians may need to individualize prophylaxis strategy based on patient-specific factors that influence the risk versus benefit equation.

Facility complexity and procedure volume are 2 well-described factors that affect SSI incidence, and may also affect choice of surgical prophylaxis regimen. To capture institutional experience and total volume of surgical procedures, which are both inversely associated with SSI incidence [27,28], we adjusted for the facility variable in multiple ways, and our effect estimate for a benefit of combination prophylaxis did not change. Similarly, region of the country—strongly associated with MRSA prevalence—was also evaluated [29], and the effect estimate remained stable. These findings support the strength of our results.

Although our cohort was large and robust, the relatively small number of MRSA-colonized patients limited our ability to quantify the impact of combination prophylaxis in this subpopulation. However, the relative risk reductions in this subpopulation did appear to be similar to the findings for the full cohort, and suggested that combination prophylaxis may be associated with reduced SSI incidence for MRSA-colonized patients undergoing cardiac but not other types of surgical procedures. In light of other studies demonstrating a benefit of vancomycin over a beta-lactam for MRSA-colonized patients [4], these data suggest that MRSA screening may be a reasonable strategy for reducing SSI (by triaging patients who receive vancomycin) and preventing AKI (by limiting the number of patients who receive 2 drugs versus 1). However, additional modeling is needed to more fully elucidate this question.

Our data are limited in several ways. First, this was a large multicenter study conducted in a VA population. When compared to other large cohorts, such as the Medicare population, veterans are predominantly male, are slightly older, tend to live in more rural areas, are more likely to be married, are more likely to have a college degree, and have slightly higher incomes [30]. Risk factors for SSI may be different in other populations, particularly for female patients. In addition, we were not able to control for some variables that may affect SSI risk, including body mass index and weight-based dosing of prophylactic regimens. However, the VA electronic health record and integrated healthcare system augmented the robustness of our data; missing data were rare. In addition, a national VA mandate requires facilities to collect MRSA colonization data for admitted patients [31]; availability of these screening results provided essential information for evaluating the association between preoperative MRSA colonization status and choice of prophylactic regimen.

A second limitation is that we were not able to evaluate all potential adverse outcomes following receipt of combination prophylaxis. In particular, we were not able to determine the effect of institutionalizing combination prophylaxis on the incidence of antimicrobial resistance, although longer durations of therapy are known to worsen this outcome [32,33]. Third, particularly for the AKI outcome, we were limited by a lack of information about potential confounding factors, such as duration of procedure and receipt of other nephrotoxic drugs; further in-depth analysis of AKI following surgery with a controlled clinical trial is needed. Fourth, we did not have information about late postoperative SSIs that may occur outside of the 30-day VASQIP review. However, most SSIs occur between 5 and 10 days postoperatively, and the vast majority occur during a 30-day window period.

Finally, this was a cohort study rather than a randomized controlled trial, which may be open to unmeasured confounding. However, for rare outcomes, this approach is robust, and randomized trials are not practical given the number of patients that would need to be included. Our study is additionally strengthened by application of a propensity score adjustment and by considering both potential positive and negative impacts of the intervention—key elements of high-quality comparative effectiveness research [34]. The data are also enriched by our ability to correlate antimicrobial prophylaxis with microbiological SSI outcomes at the patient level. The ability to account for patient- and facility-level factors in this national population and the availability of manually validated exposure and outcome variables further strengthen the robustness of our study design.

In summary, for cardiac surgery patients, combination prophylaxis was associated with a significant reduction in postoperative surgical infections, but was also associated with an increase in postoperative AKI; the risk–benefit profile varied depending upon the patient’s preoperative MRSA colonization status. There was no association between receipt of combination prophylaxis and SSI reduction for other types of surgical procedures, but the associated harm of increased postoperative AKI persisted. Future studies are needed to evaluate the utility of MRSA screening protocols for optimizing and individualizing surgical prophylaxis regimens.

## Supporting information

S1 STROBE Checklist STROBE checklist. (PDF)

S1 IRB Protocol A priori analysis plan submitted to and approved by IRB. (DOCX)

S1 Table Procedure codes included in the cohort. (DOCX)

S2 Table Relative risks corresponding to the effect of antibiotic regimen on specific 30-day surgical site infection type (superficial or deep/organ space) incidence. (DOCX)

S3 Table Adjusted relative risks corresponding to the effect of antibiotic regimen on surgical site infection incidence, including VA-facility-level variables in the model. (DOCX)

## Abbreviations

AKI: acute kidney injury
aRR: adjusted risk ratio
ASA: American Society of Anesthesiologists
CDI: *Clostridium difficile* infection
cRR: crude risk ratio
EPRP: External Peer Review Program
IRB: institutional review board
MRSA: methicillin-resistant *Staphylococcus aureus*
MSSA: methicillin-susceptible *Staphylococcus aureus*
NNH: number needed to harm
NNT: number needed to treat
PS: propensity score
RD: risk difference
RR: risk ratio
SSI: surgical site infection
VA: Veterans Affairs
VASQIP: Veterans Affairs Surgical Quality Improvement Program
