# Supplementary material for: Risk of surgical site infection, acute kidney injury, and Clostridium difficile infection following antibiotic prophylaxis with vancomycin plus a beta-lactam versus either drug alone: A national propensity-score-adjusted retrospective cohort study
Source: PLoS Med. 2017 Jul 10;14(7):e1002340. doi: 10.1371/journal.pmed.1002340 (PMC5503171; doi:10.1371/journal.pmed.1002340)
Supplement: S1 IRB Protocol — (DOCX) [file pmed.1002340.s002.docx]

**IRB Protocol; initially approved September 2013**

**Data Analysis:** VINCI will serve as the portal for all data building and analyses. Standard workspace is provisioned in VINCI so that the study has its own project site where multiple people can collaborate using a common set of software tools and files. The analysis plan encompasses multiple steps including data inspection, bivariable analyses, multivariable regression analysis, and analysis of important effect modifiers.

- First, **data distributions** will be examined for outliers, invalid codes, and missing values. Every effort will be made to ensure complete data. However, patient admissions that are missing microbiology or pharmacy data will be excluded since this is necessary to determine exposures and outcomes.
- Second, **univariable and bivariable** analyses will be performed.^75^ In short, bivariable analyses will evaluate the relationship between patient risk factors, potential confounders and each outcome. Analyses will use the chi-square test, Fisher’s exact test, Students t-test or the Wilcoxon Rank Sum test as appropriate. The Breslow-Day test will be used to assess the effect modification of MRSA carrier status on the relationship between prophylaxis and SSI. Variables that are significantly related to patient outcomes will be candidate variables for multivariable regression analyses.
- Third, multivariable **Poisson regression analysis** with a sandwich error term will be used to calculate adjusted relative risks with 95% confidence intervals and assess the association between the pre-operative MRSA nasal status, receipt of vancomycin surgical prophylaxis, and the outcomes of interest. Poisson regression analysis will be used in order to determine how MRSA nasal status and vancomycin prophylaxis influence the rate of SSI and was the recommended modeling approach used in our recent publication on this topic (Gupta et al, ICHE). Models will be estimated conditional on the admitting hospital to control for variations in practice patterns across VA hospitals and for the clustering of patients within hospitals. Additionally, to further adjust for residual confounding due to measured differences between receipt of vancomycin versus other antibiotics, **a propensity score for the probability of receipt of vancomycin will be created** and added to the final model.
- Fourth, in addition to the propensity score, we will use **instrumental variable analysis** to account for unmeasured bias.([13](#_ENREF_13)) This combined approach has been used in high impact studies on surgical and infection-related outcomes ([14](#_ENREF_14)) and it is an approach we are using in our other funded studies of MRSA infections (Schweizer CDA).([15](#_ENREF_15)) We will use surgical facility as an instrumental variable which has a high correlation with the surgical prophylaxis regimen chosen and no independent effect on SSI risk. We will also evaluate VISN as an instrumental variable to evaluate larger geographic effects. We will use the IVREG procedure in STATA software for the instrumental variable models. We will perform sensitivity analyses to determine the best model.
- Fifth, the SSI risk in patients receiving vancomycin who are positive for nasal MRSA pre-operatively compared to those who are negative will be analyzed to assess whether the pre-operative MRSA nasal status is an effect modifier of the association between SSI risk and surgical antimicrobial choice. This comparison will be done in two ways. The Breslow Day test will be used to compare unadjusted effect sizes to determine effect modification. Additionally, an interaction term including both MRSA nasal status and receipt of vancomycin will be added to each multivariable Poisson regression model to assess the effect modification adjusting for potential confounders.

**Power and Sample Size**: The EPRP process reviews approximately 30,000 procedures per year. The compliance rate with nasal MRSA screening on a national level rose from 82% to 96% during 2008-2010. Thus, we anticipate that approximately 24,600 to 28,800 of EPRP assessed patients will also have a nasal MRSA screen result available in the 0 to 31 days prior to surgery. Thus our sample size will be approximately 26,000 patients per year and 130,000 over the 5 years. Based on our previous work, approximately 7% -10% of patients will be positive for pre-operative nasal MRSA, resulting in approximately 2600 patients per year and 13,000 in total who are MRSA positive pre-operatively. If the use of vancomycin reduces the risk among MRSA positive patients by 0.60 fold (Gupta K, ICHE 2011), we will have at least 90% power with an alpha level of .05 to detect a statistically significant difference (defined as p<.05) in SSI risk from 2.5% in vancomycin un-exposed patients to 1.5% in vancomycin exposed patients.

**Aim 2 Evaluate the comparative effectiveness of vancomycin alone vs. vancomycin plus a β-lactam as surgical prophylaxis in prevention of SSI**

The population to be studied will be the same as the cohort assembled for Aim 1. Patients with MRSA nasal screening results available in the 31 days prior to surgery and with surgical prophylaxis regimen information available from the EPRP database will be included in the study. Although the comparison in regimens is most applicable to patients known to have MRSA (the group in whom vancomycin would be recommended as part of the surgical regimen), some providers use vancomycin even in non-MRSA colonized patients. Thus, MRSA nasal status will not be used to determine eligibility for the analysis, but will be used as a stratification variable.

**Data Sources and Data elements** are as described above in Aim 1. In addition, we will capture the reasons for vancomycin administration from EPRP and evaluate this variable as an effect modifier of the effectiveness of vancomycin alone vs. a 2-drug regimen in patients who do not have MRSA (and thus do not automatically meet criteria for getting vancomycin). This will allow for evaluation of criteria such as being high risk due to coming from an inpatient or nursing home facility or due to a local high prevalence rate of MRSA. The standardized method of manual data collection for EPRP allows for a robust analysis of "perceived risk" of MRSA that would not be captured in routine administrative or laboratory databases.

**Data Analysis:** First, data distributions will be reviewed. Then, bivariable and univariable analyses will be conducted. Demographic and clinical categorical data will be analyzed using a *X^2^* or Fisher’s exact test. A student’s *t*-test or Wilcoxon rank-sum test will be used for continuous variables of interest.

The propensity score will be used to adjust for risk of vancomycin exposure.([16-19](#_ENREF_16)) Wald statistics, parameter coefficients, and likelihood ratio tests will be used to guide model development, according Hosmer and Lemeshow methods.([16](#_ENREF_16)) Propensity score assumptions will be assessed along with model fit using Hosmer-Lemeshow goodness-of-fit, which examines observed and expected frequencies within deciles of risk.([18-20](#_ENREF_18)) Model discrimination, or the ability to differentiate between patients receiving vancomycin alone vs. the 2-drug regimen, will be evaluated with the area under the receiver operating characteristic curve.([16](#_ENREF_16)) Multicollinearity will be assessed using the correlation matrix of the final model and variance inflation factor values.([16](#_ENREF_16))

Cox proportional hazard models will assess the hazard (risk) of SSI among patients who receive vancomycin compared to patients who receive the 2-drug regimen, statistically adjusting for the propensity score. Cox proportional hazard models will be used to perform time to event analyses and model assumptions, including that of proportionality, will be evaluated with formal tests and graphical displays.([21](#_ENREF_21)) From the final adjusted models, the hazard ratios and corresponding 95% confidence intervals will be determined. All analyses will be performed using SAS (SAS Institute Inc., Cary, NC, Version 9.1.3).

**Power and Sample Size:** The EPRP process reviews approximately 30,000 procedures per year for a total of 150,000 procedures during the study period. According to our prior study (Gupta ICHE 2011) in one VA hospital, 4% of surgical patients received prophylaxis with vancomycin alone and 2% of patients received prophylaxis with vancomycin plus a beta-lactam antibiotic. Our study will have 85% power to detect a 2 fold difference in rates of SSI between patients who receive vancomycin (proportion with SSI=1.5%) and patients who receive vancomycin plus a beta lactam antibiotic (proportion with SSI=0.07%).
