# Supplementary material for: Risk of surgical site infection, acute kidney injury, and Clostridium difficile infection following antibiotic prophylaxis with vancomycin plus a beta-lactam versus either drug alone: A national propensity-score-adjusted retrospective cohort study
Source: PLoS Med. 2017 Jul 10;14(7):e1002340. doi: 10.1371/journal.pmed.1002340 (PMC5503171; doi:10.1371/journal.pmed.1002340)
Supplement: S1 Table — (DOCX) [file pmed.1002340.s003.docx]

**S1. Table. Procedure Codes Included in the Cohort.**

| Cardiac Surgery Cases |  |
| --- | --- |
| Coronary Artery Bypass Grafting (CABG) | 36.1, 36.11, 36.12, 36.13, 36.14, 36.15, 36.16, 36.17, 36.19 |
| Other Cardiac Surgery | 35.11, 35.12, 35.13, 35.14, 35.20, 35.21, 35.22, 35.23, 35.24, 35.25, 35.26, 35.27, 35.28, 35.32, 35.33, 35.39, 35.42, 35.51, 35.53, 35.54, 35.61, 35.72, 35.73, 35.82, 35.93, 35.98, 35.99 |
| Colorectal Cases | 17.31, 17.32, 17.33, 17.34, 17.35, 17.36, 17.39, 45.03, 45.49, 45.50, 45.71, 45.72, 45.73, 45.74, 45.75, 45.76, 45.79, 45.82, 45.90, 45.92, 45.93, 45.94, 45.95, 46.03, 46.04, 46.10, 46.13, 46.76, 46.94, 48.50, 48.61, 48.62, 48.63, 48.64, 48.65, 48.69 |
| Hysterectomies | 68.31, 68.49, 68.51, 68.59, 68.69, 68.79 |
| Orthopedic Surgery Cases |  |
| Hip Procedures | 81.51, 81.52 |
| Knee Procedures | 81.54 |
| Vascular Surgery Cases | 38.14, 38.16, 38.18, 38.34, 38.36, 38.37, 38.44, 38.48, 38.49, 38.64, 39.25, 39.26, 39.29 |
