# Supplementary material for: Risk of surgical site infection, acute kidney injury, and Clostridium difficile infection following antibiotic prophylaxis with vancomycin plus a beta-lactam versus either drug alone: A national propensity-score-adjusted retrospective cohort study
Source: PLoS Med. 2017 Jul 10;14(7):e1002340. doi: 10.1371/journal.pmed.1002340 (PMC5503171; doi:10.1371/journal.pmed.1002340)
Supplement: S2 Table — (DOCX) [file pmed.1002340.s004.docx]

**S2. Table:** Relative risks corresponding to the effect of antibiotic regimen on specific 30-day surgical site infection type (superficial or deep/organ space) incidence.^a,b,c,d^

|  | #Superficial; #Analyzed | RR, Superficial (95% CI) | #Deep/Organ Space; #Analyzed | RR, Deep/Organ Space (95% CI) |
| --- | --- | --- | --- | --- |
| ***Cardiac*** |  |  |  |  |
| Crude | 137; 19668 | 0.73 (0.51, 1.06) | 119; 19650 | 0.54 (0.35, 0.83) |
| PS-adjusted | 122; 18516 | 0.72 (0.49, 1.07) | 108; 18502 | 0.50 (0.31, 0.79) |
| *MRSA Colonized* |  |  |  |  |
| Crude | 6; 440 | 1.41 (0.17, 11.96) | 6; 440 | 0.29 (0.06, 1.42) |
| PS-adjusted | 5; 415 | 1.18 (0.13, 10.39) | 6; 416 | 0.31 (0.06, 1.50)^e^ |
| *MRSA- /Unknown* |  |  |  |  |
| Crude | 112; 16730 | 0.67 (0.45, 1.00) | 92; 16710 | 0.54 (0.34, 0.87) |
| PS-adjusted | 105; 15903 | 0.68 (0.45, 1.03) | 86; 15884 | 0.50 (0.30, 0.81) |
| ***Orthopedic*** |  |  |  |  |
| Crude | 244; 33657 | 1.20 (0.85, 1.68) | 191; 33604 | 1.05 (0.70, 1.57) |
| PS-adjusted | 210; 31688 | 1.08 (0.74, 1.58) | 172; 31650 | 1.11 (0.73, 1.68) |
| *MRSA Colonized* |  |  |  |  |
| Crude | 12; 425 | 1.26 (0.38, 4.11) | 1; 414 | - ^f^ |
| PS-adjusted | 9; 398 | 1.05 (0.26, 4.28) | 1; 390 | - ^f^ |
| *MRSA- /Unknown* |  |  |  |  |
| Crude | 206; 30743 | 1.08 (0.74, 1.57) | 165; 30702 | 1.15 (0.76, 1.73) |
| PS-adjusted | 192; 29213 | 1.05 (0.71, 1.55) | 155; 29176 | 1.13 (0.74, 1.73) |
| ***Vascular*** |  |  |  |  |
| Crude | 444; 7243 | 1.00 (0.72, 1.40) | 162; 6961 | 1.19 (0.70, 2.01) |
| PS-adjusted | 399; 6739 | 0.98 (0.69, 1.39) | 143; 6483 | 1.02 (0.57, 1.84) |
| *MRSA Colonized* |  |  |  |  |
| Crude | 4; 89 | 0.45 (0.05, 4.14) | 7; 92 | 0.54 (0.11, 2.66) |
| PS-adjusted | 4; 81 | 0.64 (0.07, 5.61) | 6; 83 | 0.73 (0.14, 3.83) |
| *MRSA- /Unknown* |  |  |  |  |
| Crude | 381; 6261 | 1.06 (0.75, 1.48) | 127; 6007 | 1.23 (0.70, 2.16) |
| PS-adjusted | 350; 5829 | 1.00 (0.70, 1.43) | 115; 5594 | 1.09 (0.59, 2.01) |
| ***Colorectal*** |  |  |  |  |
| Crude | 711; 6915 | 1.10 (0.72, 1.67) | 401; 6605 | 0.98 (0.53, 1.80) |
| PS-adjusted | 642; 6315 | 1.10 (0.71, 1.71) | 380; 6053 | 1.02 (0.56, 1.88) |

^a^ RR = Relative Risk, CI = Confidence Interval, PS = Propensity Score.

^b^ Exposure reference groups were either antibiotic for the cardiac, orthopedic and vascular full cohort models; vancomycin alone for all MRSA colonized-only models; beta-lactam alone for all MRSA- /Unknown-only and colorectal full cohort models.

^c^ RR rounded to two decimal places.

^d^ Via the pure propensity score, all models were adjusted for age, diabetes status, ASA score, mupirocin status and smoking status. The full cohort models also were adjusted for MRSA colonization status.

^e^ Poisson distribution with log link and robust standard errors from generalized estimating equations applied.

^f^ Only one event occurred in this sub-group.
