# Supplementary material for: Risk of surgical site infection, acute kidney injury, and Clostridium difficile infection following antibiotic prophylaxis with vancomycin plus a beta-lactam versus either drug alone: A national propensity-score-adjusted retrospective cohort study
Source: PLoS Med. 2017 Jul 10;14(7):e1002340. doi: 10.1371/journal.pmed.1002340 (PMC5503171; doi:10.1371/journal.pmed.1002340)
Supplement: S3 Table — (DOCX) [file pmed.1002340.s005.docx]

**S3. Table:** Adjusted relative risks corresponding to the effect of antibiotic regimen on surgical site infection incidence, including VA facility-level variables in the model^a,b,c,d^

|  | #SSI; #Analyzed | aRR (95% CI) |
| --- | --- | --- |
| ***Cardiac*** |  |  |
| +Region | 232; 17829 | 0.65 (0.49, 0.88) |
| +Facility Complexity | 235; 18312 | 0.61 (0.45, 0.81) |
| +Facility Volume Quartile | 230; 18345 | 0.61 (0.46, 0.83) |
| ***Orthopedic*** |  |  |
| +Region | 296; 25030 | 1.20 (0.89, 1.62) |
| +Facility Complexity | 394; 31525 | 1.13 (0.85, 1.48) |
| +Facility Volume Quartile | 386; 30846 | 1.05 (0.79, 1.39) |
| ***Vascular*** |  |  |
| +Region | 526; 6793 | 1.06 (0.79, 1.42) |
| +Facility Complexity | 550; 6718 | 0.97 (0.72, 1.30) |
| +Facility Volume Quartile | 541; 6787 | 1.00 (0.74, 1.35) |
| ***Colorectal*** |  |  |
| +Region | 979; 6303 | 1.08 (0.77, 1.52) |
| +Facility Complexity | 975; 6350 | 0.93 (0.65, 1.34) |
| +Facility Volume Quartile | 1003; 6330 | 1.04 (0.73, 1.46) |

^a^ For each surgery sub-cohort, the original propensity-score adjusted full cohort model was reconstructed to include region, facility complexity or facility volume quartile (three separate models).

^b^ SSI = Surgical Site Infection, RR = Adjusted Relative Risk, CI = Confidence Interval.

^c^ Exposure reference groups were either antibiotic for the cardiac, orthopedic and vascular models; beta-lactam alone for the colorectal models.

^d^ A maximum of 2.5% of observations were deleted from any of the logistic regression models that were used to generate propensity scores due to missing outcome or explanatory variable data.
